# A systematic review and meta-analysis of the systemic immune-inflammation index (SII) in rheumatic diseases

**DOI:** 10.3389/fimmu.2026.1896434

**Published:** 2026-07-13

**Authors:** Arduino A. Mangoni, Angelo Zinellu

**Affiliations:** 1 Discipline of Clinical Pharmacology, College of Medicine and Public Health, Flinders University, Adelaide, SA, Australia; 2 Department of Clinical Pharmacology, Flinders Medical Centre, Southern Adelaide Local Health Network, Adelaide, SA, Australia; 3 Department of Biomedical Sciences, University of Sassari, Sassari, Italy

**Keywords:** autoimmunity, biomarkers, inflammation, rheumatic diseases, SII, systemic immune-inflammation index

## Abstract

**Introduction:**

Accessible biomarkers that reflect immune-inflammatory activity may support the diagnosis and monitoring of patients with rheumatic diseases (RDs). We conducted a systematic review and meta-analysis to investigate the systemic immune-inflammation index (SII), a hematological index derived from standard full blood count parameters and calculated as (neutrophil count x platelet count)/lymphocyte count, as a potential adjunctive biomarker in RDs.

**Methods:**

We searched PubMed, Web of Science, and Scopus from inception to 27 May 2026 for studies investigating the SII in patients with RDs and healthy controls. We assessed the risk of bias using the Joanna Briggs Institute Critical Appraisal Checklist for analytical studies and the certainty of evidence using the Grading of Recommendations, Assessment, Development and Evaluation Working Group system.

**Results:**

In 52 studies (6,903 patients with RDs, mean age 47.9 years, 55% female, and 6,724 healthy controls, mean age 44.6 years, 45% female), compared with controls, RDs patients had significantly higher SII values (standardized mean difference, SMD = 0.83, 95% CI 0.69 to 0.97; p<0.001; I^2^ = 91.6%, p<0.001; very low certainty of evidence). In meta-regression analysis, no significant associations were observed between the effect size and age, male-to-female ratio, year of publication, sample size, mean disease duration, C-reactive protein, erythrocyte sedimentation rate, active-to-remission patient ratio, or use of antirheumatic drugs. In subgroup analysis, the pooled SMD was statistically significant in studies conducted in Asia, Africa, and Europe, with substantially lower heterogeneity in European studies. The pooled SMD was also statistically significant across most RD categories, except fibromyalgia and connective tissue disease.

**Discussion:**

These findings indicate that the SII is higher in RD case groups than in healthy controls and support further evaluation of the SII as an adjunct to established diagnostic criteria, disease activity scores, and serological biomarkers. Large, appropriately designed prospective studies in diverse RD populations are warranted to define clinically useful thresholds and determine whether the SII improves diagnostic or prognostic performance.

**Systematic review registration:**

https://www.crd.york.ac.uk/PROSPERO/, identifier CRD420261409915.

## Introduction

Rheumatic diseases (RDs) encompass a broad spectrum of immune-mediated conditions, including predominantly autoimmune disorders such as rheumatoid arthritis (RA), mixed autoimmune-autoinflammatory diseases such as Behcet’s disease (BD), and autoinflammatory syndromes such as familial Mediterranean fever (FMF). Prompt recognition and treatment are clinically important because persistent inflammation can lead to pain, organ involvement, functional impairment, irreversible tissue damage, reduced quality of life, and increased healthcare use (1–9). In practice, however, early diagnosis remains difficult as manifestations may be intermittent or non-specific, serology can be negative, imaging findings may lag behind symptoms, and non-specialists often encounter patients before disease-defining features have fully evolved (10–15). This has stimulated interest in biomarkers that are inexpensive, reproducible, widely available, and interpretable together with clinical findings. Such tests are particularly valuable in treat-to-target strategies, where timely assessment of inflammatory activity informs referral, treatment escalation, response monitoring, and the prevention of damage (16–20).

The routine assessment of RDs already relies heavily on circulating inflammatory biomarkers, particularly C-reactive protein (CRP), erythrocyte sedimentation rate (ESR), and ferritin (14, 21–24). These tests are useful because they broadly reflect acute-phase responses, but they lack disease specificity and may be influenced by infection, age, sex, adiposity, anemia, hepatic function, and drug exposure. Moreover, normal CRP or ESR values do not exclude active disease in several RDs, whereas elevated values do not necessarily indicate immune-mediated rheumatic inflammation (21, 25–27). Consequently, investigators have increasingly examined blood cell-derived markers, including neutrophil, platelet, and lymphocyte counts and ratios, as pragmatic surrogates for systemic inflammation and immune regulation (28–34). Within this family of indices, the systemic immune-inflammation index, commonly calculated as SII = (neutrophil count x platelet count)/lymphocyte count, has been studied extensively in cancer (35, 36), cardiovascular disease (37), liver disease (38), and coronavirus disease 2019 (COVID-19) (39).

The biological rationale for evaluating the SII in RDs rests on the contribution of each of its cellular components to immune-mediated pathology. Neutrophils participate in cytokine release, oxidative responses, tissue recruitment, antigen presentation, and neutrophil extracellular trap formation, processes relevant to RA, SLE, vasculitis, gout, and autoinflammatory disease (40, 41). Platelets are increasingly recognized as immune-effector cells that interact with leukocytes and endothelium, release cytokines and microparticles, and promote thrombo-inflammatory pathways (42, 43). Lymphocyte counts, by contrast, may reflect adaptive immune activation, redistribution, treatment effects, or disease-associated lymphopenia (44, 45). By integrating these three cell types, the SII may provide a composite measure of systemic immune-inflammatory imbalance rather than a simple acute-phase reaction. This property is particularly relevant in RDs, where inflammatory activity, immune dysregulation, endothelial activation, and treatment effects coexist and vary across disease phenotypes and stages (46).

An earlier systematic review and meta-analysis of the SII across immunological diseases found higher SII values in affected patients than in healthy controls and suggested diagnostic potential across a heterogeneous disease spectrum (47), Since that search, however, the RD-specific literature has expanded substantially, with additional studies across RA, SLE, spondyloarthritis, vasculitis, gout, Sjögren’s syndrome, systemic sclerosis, fibromyalgia, and osteoarthritis. A disease-focused synthesis is therefore warranted to clarify whether the association is consistent across RD phenotypes, whether heterogeneity can be explained by demographic or clinical characteristics, and whether the existing evidence is sufficiently robust to justify clinical translation. We therefore conducted an updated systematic review and meta-analysis of studies comparing SII values in adults with RDs and healthy controls and examined whether effect sizes varied according to RD type, disease duration, CRP, ESR, active-to-remission patient ratio, use of disease-modifying antirheumatic drugs (DMARDs), and geographical region.

## Materials and methods

### Search strategy, screening, and study selection

We systematically searched PubMed, Web of Science, and Scopus from inception to 27 May 2026 using terms for SII (“SII” OR “systemic inflammatory index” OR “systemic immune-inflammation index”) combined with terms for RDs (“rheumatic diseases” OR “rheumatoid arthritis” OR “psoriatic arthritis” OR “reactive arthritis” OR “ankylosing spondylitis” OR “systemic lupus erythematosus” OR “systemic sclerosis” OR “scleroderma” OR “Sjögren’s syndrome” OR “connective tissue diseases” OR “vasculitis” OR “Behçet’s disease” OR “idiopathic inflammatory myositis” OR “polymyositis” OR “dermatomyositis” OR “gout” OR “pseudogout” OR “systemic vasculitis” OR “ANCA-associated vasculitis” OR “Takayasu arteritis” OR “polyarteritis nodosa” OR “osteoarthritis” OR “fibromyalgia” OR “granulomatous polyangiitis” OR “Wegener’s granulomatosis” OR “IgA vasculitis” OR “Henoch-Schönlein purpura” OR “familial Mediterranean fever” OR “polymyalgia rheumatica” OR “temporal arteritis” OR “giant cell arteritis”) (**Supplementary Table 1**).

Two investigators independently screened the abstracts to determine relevance and, if appropriate, the full articles. Inclusion criteria were: (i) investigation of the SII in patients with RD diagnosed according to accepted guidelines and healthy controls in a case-control study, (ii) recruitment of adult participants, and (iii) availability of the full text of the publication in the English language. Exclusion criteria were: (i) non-case-control studies, (ii) participants <18 years, and (iii) cellular or animal studies. References from the retrieved articles were also searched for additional studies. The following parameters were independently extracted: year of publication, first author, country where the study was conducted, sample size, age, sex, disease type, CRP, ESR, and use of disease-modifying antirheumatic drugs (DMARDs). Any disagreements between the investigators were resolved through discussion and consensus.

The risk of bias was evaluated using the Joanna Briggs Institute (JBI) Critical Appraisal Checklist for analytical studies (48). Meeting ≥75%, ≥50 and <75%, and <50% of the checklist items indicated low, moderate, and high risk, respectively. The certainty of evidence was evaluated using the Grading of Recommendations, Assessment, Development, and Evaluation (GRADE) Working Group system. GRADE takes into account the study design (randomized vs. observational), the risk of bias, the presence of unexplained heterogeneity, the indirectness of evidence, the imprecision of results (sample size, 95% confidence interval width, and threshold crossing), the effect size (small, standard mean difference (SMD) <0.5, moderate, SMD 0.5-0.8, and large, SMD >0.8) (49), and the probability of publication bias (50, 51). The study adhered to the Preferred Reporting Items for Systematic Reviews and Meta-Analyses (PRISMA) 2020 statement (**Supplementary Table 1**) (52). The protocol was registered in the International Prospective Register of Systematic Reviews (PROSPERO, CRD420261409915).

### Statistical analysis

Standardized mean differences (SMDs) and 95% confidence intervals (CIs) were calculated to generate forest plots for continuous data and to assess differences in the SII between patients with rheumatic diseases and healthy controls. A p-value of <0.05 was considered statistically significant. When necessary, mean and standard deviation were estimated from median and interquartile ranges, or from medians and ranges (53). The heterogeneity of SMD across studies was evaluated using the Q statistic (significance level at p<0.10). Heterogeneity was classified as low when I^2^ ≤ 25%, moderate when 25% < I^2^ < 75%, and high when I^2^ ≥ 75% (54, 55). For meta-analyses with substantial heterogeneity, a random-effects model based on the inverse-variance method was employed. Sensitivity analysis was conducted to examine the influence of each individual study on the overall risk estimate by sequentially excluding one study (56). To assess potential publication bias, the relationship between study size and effect magnitude was analyzed using Begg’s adjusted rank correlation test and Egger’s regression asymmetry test at a significance level of p<0.05 (57, 58). The Duval and Tweedie “trim and fill” procedure was performed to further examine and potentially correct for any publication bias (59). Univariate meta-regression analyses were conducted to investigate associations between the effect size and the following parameters: year of publication, age, gender, sample size, mean disease duration, CRP, ESR, active-to-remission patient ratio, or DMARD administration. Statistical analyses were performed using Stata 18 (StataCorp LLC, College Station, TX, USA).

## Results

### Systematic search and study characteristics

**Figure 1:**
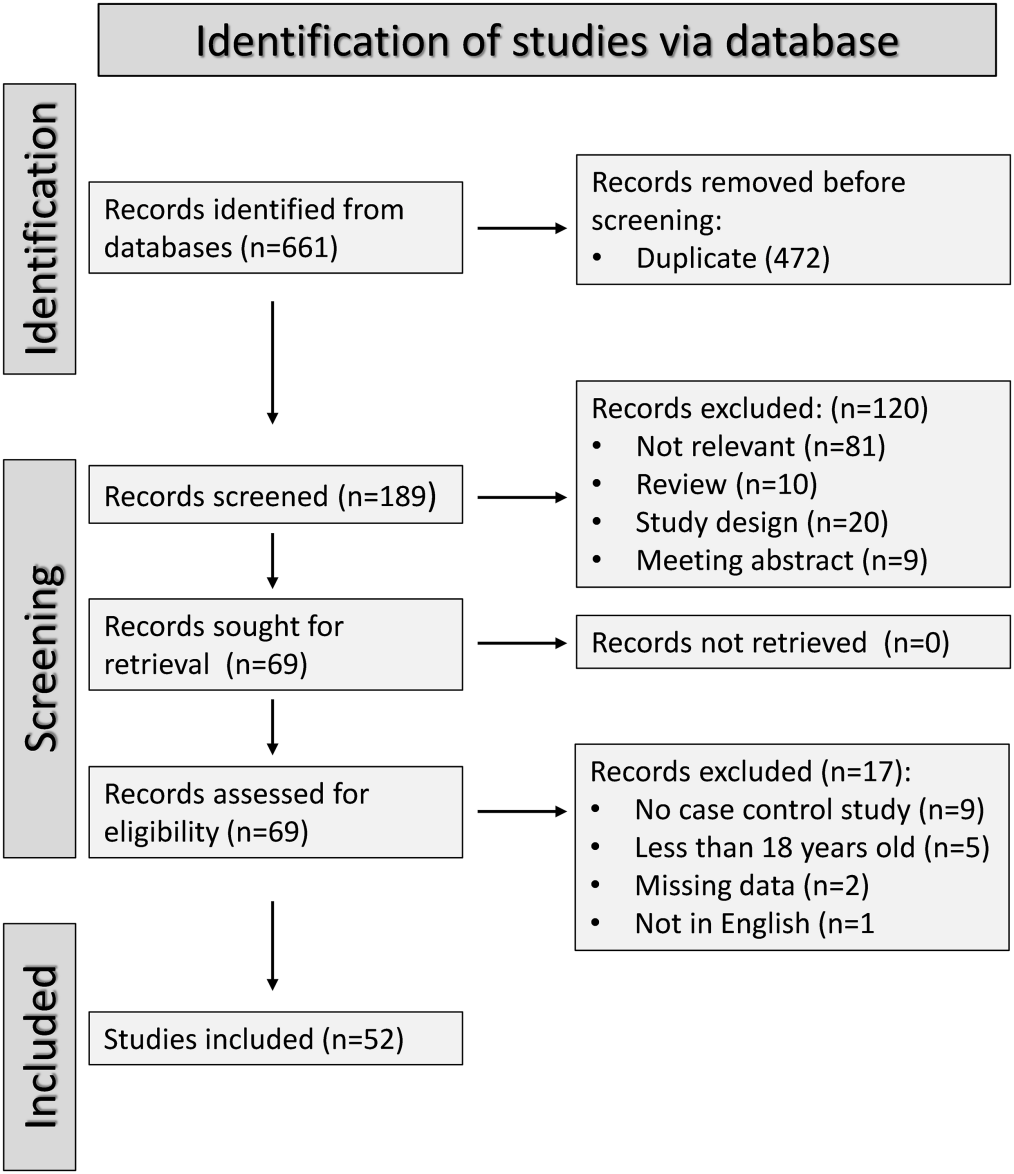
PRISMA 2020 flow diagram of screening and study selection.

**Figure 1** presents a flowchart illustrating the screening process. A total of 661 records were initially identified through database searches. After removing 472 duplicate records, 189 were screened based on their titles and abstracts. Of these, 120 records were excluded for the following reasons: 81 were off-topic, 20 had an inappropriate study design, 10 were review articles, and nine were meeting abstracts. Following a comprehensive review of the full texts of the remaining 69 articles, nine were further excluded for the absence of a control group, five for including patients younger than 18 years, two for missing data or insufficient information, and one for not being published in English. Ultimately, 52 studies, published between 2020 and 2026, were included in the meta-analysis (**Table 1**) (60–111). There was full concordance between the two investigators for the literature search and data extraction. These studies included 55 group comparisons, with 6,903 patients (mean age 47.9 years, 55% female) and 6,724 healthy controls (mean age 44.6 years, 45% female). A total of 45 studies were conducted in Asia, four in Africa, and three in Europe (**Table 1**). Fourteen study groups included individuals with rheumatoid arthritis (RA), nine with systemic lupus erythematosus (SLE), seven with ankylosing spondylitis (AS), four with gout, three with psoriatic arthritis, three with ANCA-associated vasculitis (AAV), three with osteoarthritis (OA), three with Sjögren’s syndrome (SS), three with fibromyalgia (FM), two with Behçet’s disease (BD), two with systemic sclerosis (SSc), one with connective tissue disease (CTD), and one with familial Mediterranean fever (FMF) (**Table 1**). Mean disease duration was reported in 21 studies and ranged between 2.5 and 15.7 years. The risk of bias was low in 50 studies and moderate in two (**Supplementary Table 2**). The cross-sectional design of the selected studies accounted for the low initial certainty of the evidence (level 2).

**Table 1:** Baseline characteristics and study findings.

	Controls				Patients with rheumatic diseases				Disease type
	n	Age (years)	Sex (M/F)	SII (mean ± SD)	n	Age (years)	Sex (M/F)	SII (mean ± SD)	
Ruta VM et al., 2020, Romania (60)	50	55.0	24/26	569.7 ± 326.7	18	57.6	11/7	671.6 ± 365.7	CTD
Wu J et al., 2021, China (61)	63	38.7	48/15	297.0 ± 112.0	136	34.0	118/18	491.5 ± 249.0	AS
Choe JY et al., 2022, South Korea (62)	80	58.1	5/75	386.9 ± 231.8	123	55.6	15/108	967.4 ± 598.4	RA
Kelesoglu Dincer AB et al., 2022, Turkey (63)	103	47.3	40/63	530.1 ± 204.0	106	47.7	47/59	785.7 ± 412.7	PsA
Luo Q et al., 2022, China (64)	75	33.0	55/20	412.5 ± 203.9	79	32.3	55/24	874.4 ± 781.5	AS
Taha SI et al. (a), 2022, Egypt (65)	100	77.0	49/51	573.8 ± 262.5	100	66.0	19/81	1108.5 ± 697.2	RA
Taha SI et al. (b), 2022, Egypt (65)	100	77.0	49/51	573.8 ± 262.5	100	62.4	16/84	759.9 ± 523.0	SLE
Taha SI et al. (c), 2022, Egypt (65)	100	77.0	49/51	573.8 ± 262.5	50	67.4	33/17	1434.4 ± 888.4	AS
Amirpour A et al., 2023, Iran (66)	103	43.5	90/13	350.3 ± 158.1	103	45.7	92/11	453.5 ± 358.3	SLE
Chikovani T et al., 2023, Georgia (67)	28	48.3	8/20	466.0 ± 207.8	54	52.7	6/48	861.7 ± 517.3	RA
Choe JY et al., 2023, South Korea (68)	71	60.4	0/71	409.1 ± 277.4	257	60.7	0/257	697.1 ± 579.4	RA
Dede BT et al., 2023, Turkey (69)	51	41.9	37/14	453.8 ± 453.8	104	41.3	69/35	459.6 ± 210.3	AS
Jiang Y et al., 2023, China (70)	194	42.6	194/0	394.3 ± 137.3	873	42.9	873/0	505.3 ± 255.3	Gout
Karadeniz H et al., 2023, Turkey (71)	27	42.6	12/15	484.3 ± 181.6	38	48.2	16/22	2533.5 ± 1780.4	AAV
Ozdemir A et al., 2023, Turkey (72)	76	39.7	10/66	457.5 ± 590.9	76	39.3	11/65	1159 ± 1872.0	SLE
Sariyildiz A et al., 2023, Turkey (73)	50	41.2	33/17	418.0 ± 164.8	100	45.2	68/32	603.4 ± 287.8	AS
Sugimoto E et al., 2023, Japan (74)	50	54.3	32/18	733.0 ± 595.0	47	55.5	29/18	1105.0 ± 1515.0	PsA
Tarabeih N et al., 2023, Israel (75)	519	51.9	251/268	455.0 ± 314.6	98	56.2	30/68	615.2 ± 405.5	OA
Akdogan MR et al., 2024, Turkey (76)	69	36.2	4/65	390.4 ± 131.6	68	38.9	4/64	606.9 ± 336.5	SLE
Başaran PO et al., 2024, Turkey (77)	49	45.1	28/21	580.0 ± 244.0	67	41.5	40/27	1219.4 ± 177	RA
Dervisevic A et al., 2024, Bosnia and Herzegovina (78)	31	50.6	4/27	304.5 ± 69.9	58	55.2	2/56	509.3 ± 281.7	RA
Elnemr RA et al., 2024, Egypt (79)	67	49.3	NR	439.7 ± 233.4	115	51.8	NR	645.4 ± 647.6	RA
Ergun MC et al., 2024, Turkey (80)	99	35.2	10/89	493.6 ± 233.1	141	36.9	8/133	985.4 ± 1054.6	SLE
Kılıc O et al., 2024, Turkey (81)	90	54.0	20/70	418.5 ± 197.1	116	58.0	30/86	670.6 ± 529.1	RA
Misirci S et al., 2024, Turkey (82)	71	41.7	46/25	355.8 ± 278.8	130	44.3	96/34	636.7 ± 839.1	AS
Okutan I et al., 2024, Turkey (83)	51	46.9	20/31	501.8 ± 218.8	97	54.8	25/72	855 ± 546.4	RA
Rabrenovic V a et al., 2024, Serbia (84)	23	42.9	NR	438.6 ± 169.9	66	54.9	NR	603.7 ± 362.4	SLE
Sariyildiz A et al., 2024, Turkey (85)	90	43.4	14/76	445.5 ± 197.9	502	45.2	78/424	508.3 ± 226.4	FM
Uzeli US et al., 2024, Turkey (86)	28	50.7	0/28	326.1 ± 140.8	28	51.4	0/28	1415.8 ± 385.1	SS
Zhang Y et al., 2024, China (87)	452	37.7	190/262	347.2 ± 138.6	58	44.5	45/13	640.1 ± 402.7	PsA
Aci R et al., 2025, Turkey (88)	114	25.0	37/67	582.5 ± 394.5	70	25.5	33/37	811.1 ± 491.4	BD
Baran E et al., 2025, Turkey (89)	39	55.4	16/23	621.2 ± 245	39	55.6	16/23	3550 ± 2578	AAV
Ozdogan Bircan A et al., 2025, Turkey (90)	113	50.8	3/110	564.4 ± 205.2	89	53.4	2/86	1791 ± 1150	SS
Dogan M et al., 2025, Turkey (91)	52	45.0	16/36	461.0 ± 207.0	52	50.0	13/39	580.0 ± 463.0	SS
Ecesoy V et al., 2025, Turkey (92)	54	49.7	4/50	577.8 ± 218.0	53	53.3	4/49	932.4 ± 582.5	SSc
Gunaydin EB et al., 2025, Turkey (93)	125	67.0	34/91	480.0 ± 197.7	250	68.1	57/193	488.2 ± 262.4	OA
Helbawi FM et al., 2025, Egypt (94)	20	38.2	3/17	442.1 ± 238.0	31	40.7	3/28	767.2 ± 404.1	SSc
Huang C et al., 2025, China (95)	1788	34.6	1740/48	380.9 ± 210.0	447	34.6	435/12	625.9 ± 563.2	Gout
Klisic A et al., 2025, Turkey (96)	97	38.3	42/55	4.86 ± 2.32	114	34.0	50/64	5.63 ± 2.79	FMF
Koca N et al., 2025, Turkey (97)	277	45.6	30/247	2.08 ± 0.77	294	48.1	14/280	3.96 ± 1.99	FM
Kosehasanogullari M et al., 2025, Turkey (98)	84	45.4	NR	546.5 ± 313.7	85	46.6	NR	516.4 ± 183.4	FM
Misirci S et al., 2025, Turkey (99)	69	47.5	18/51	352.5 ± 77.1	104	48.5	25/79	781.3 ± 348.6	RA
Rapapa KA et al., 2025, China (100)	171	34.3	NR	388.8 ± 195.5	171	34.3	126/45	575.6 ± 378.8	AS
Uysal A et al., 2025, Turkey (101)	50	60.9	8/42	494.1 ± 221.8	117	60.5	20/97	681.6 ± 326.5	OA
Wu H et al., 2025, China (102)	90	60.8	14/76	444.9 ± 190.7	132	58.3	31/101	628.3 ± 374.3	RA
Yang CH et al., 2025, China (103)	91	40.4	16/75	388.6 ± 183.1	189	41.2	26/163	592.7 ± 521.2	SLE
Yigit E et al., 2025, Turkey (104)	44	58.7	19/25	381.1 ± 250.5	44	65.6	25/19	440.8 ± 270.5	Gout
Yu R et al., 2025, China (105)	65	65.6	32/33	446.6 ± 22.4	65	66.0	31/34	1813.7 ± 221.9	AAV
Zhao H et al., 2025, China (106)	48	39.2	3/45	436.4 ± 184.5	61	41.2	8/53	705.6 ± 683.7	SLE
Abdul-Sahib NS et al. (a), 2026, Iraq (107)	50	27.4	25/25	416.7 ± 135.9	50	31.2	25/25	579 ± 356.5	RA
Abdul-Sahib NS et al. (b), 2026, Iraq (107)	50	63.3	25/25	397.0 ± 201.5	50	65.0	25/25	660 ± 426.7	RA
Ashour DM et al., 2026, Egypt (108)	19	37.5	16/3	615.5 ± 222.8	39	33.7	33/6	765.5 ± 436.7	BD
Li M et al., 2026, China (109)	20	38.3	2/18	328.3 ± 179.1	100	40.1	4/96	514.2 ± 312.9	SLE
Tuzun Z et al., 2026, Turkey (110)	119	54.1	86/33	384.0 ± 123.2	119	56.7	96/23	492.8 ± 234.3	Gout
Zhang J et al., 2026, China (111)	115	59.7	14/101	438.8 ± 178.0	230	60.9	22/208	711.4 ± 535.9	RA

AAV, ANCA-associated vasculitis; AS, ankylosing spondylitis; BD, Behcet’s disease; CTD, connective tissue disease; F, female; FM, fibromyalgia; FMF, familial Mediterranean fever; M, male; NR, not reported; OA, osteoarthritis; PsA, psoriatic arthritis; RA, rheumatoid arthritis; SD, standard deviation; SII, systemic immune-inflammation index; SLE, systemic lupus erythematosus; SS, Sjögren’s syndrome; SSc, systemic sclerosis.

### Results of individual studies and syntheses

**Figure 2:**
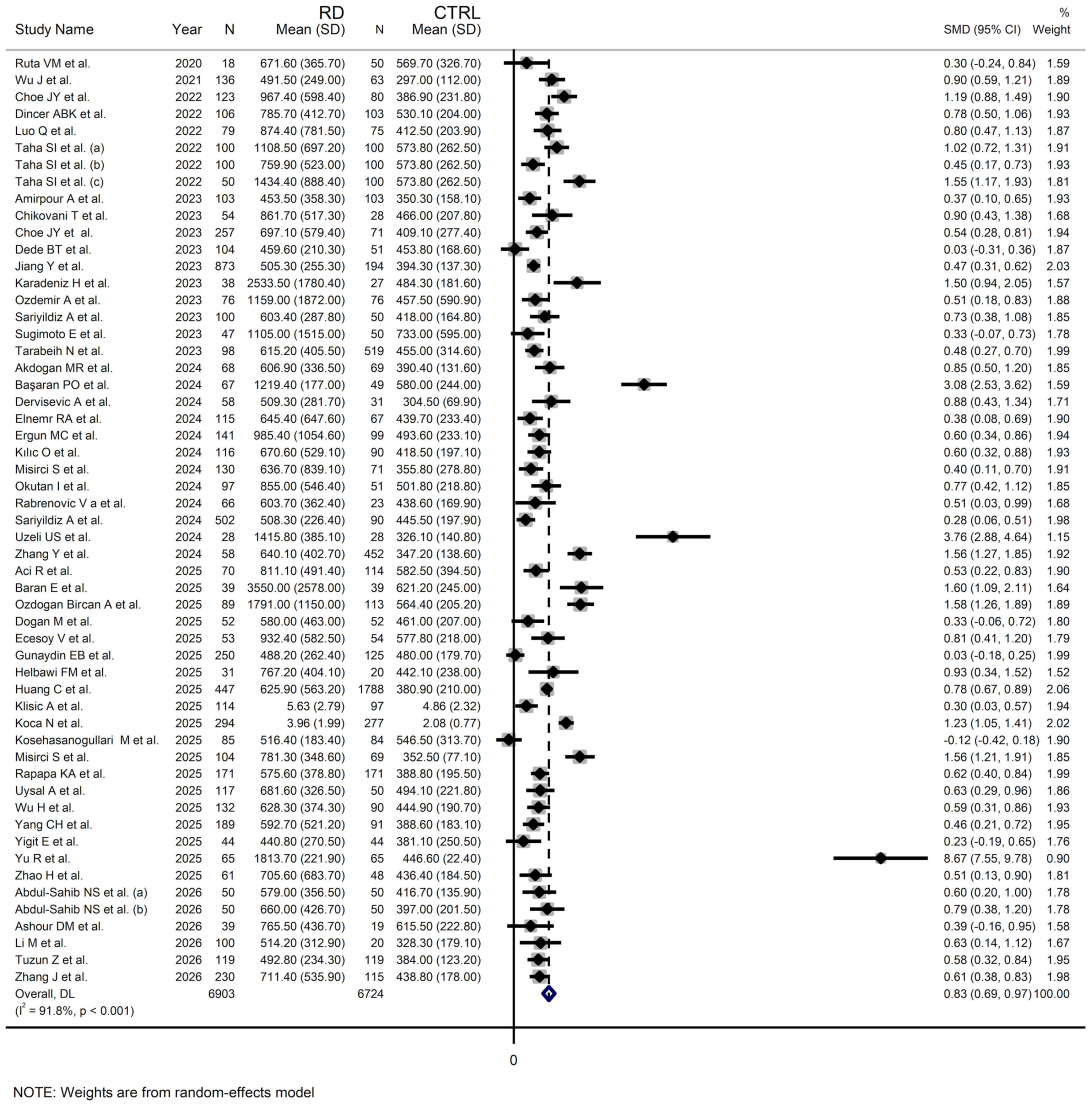
Forest plot of the SII in patients with rheumatic diseases and healthy controls.

**Figure 3:**
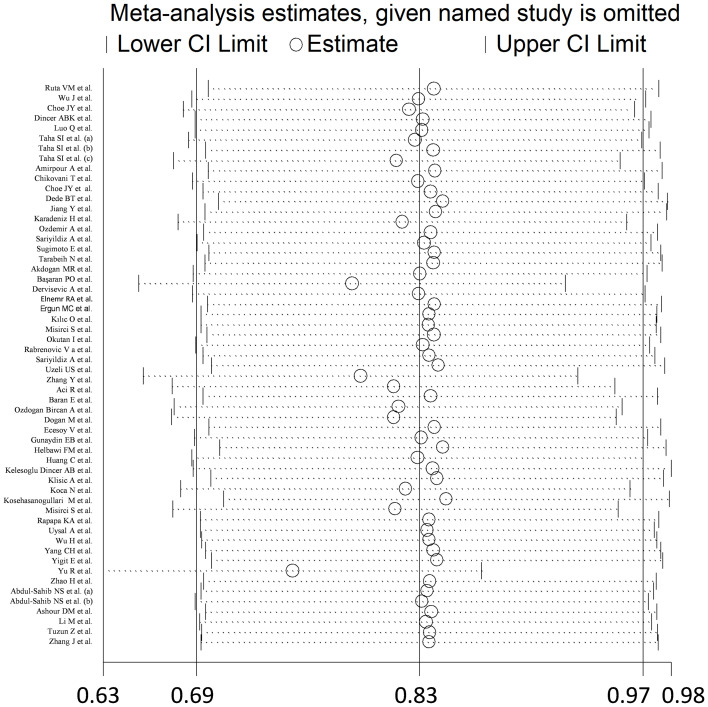
Sensitivity analysis of the association between the SII and rheumatic diseases.

The forest plot of SII values in healthy controls and patients with rheumatic diseases is shown in **Figure 2**. Elevated heterogeneity between studies was observed (I^2^ = 91.6%, p<0.001), thus random-effects models were used. Overall, pooled results showed a significantly higher SII in patients with rheumatic diseases than in controls (SMD = 0.83, 95% CI 0.69 to 0.97; p<0.001). Sensitivity analysis showed that the corresponding pooled SMD values were not significantly altered when any single study was omitted (effect sizes ranged from 0.63 to 0.84; **Figure 3**).

### Publication bias

**Figure 4:**
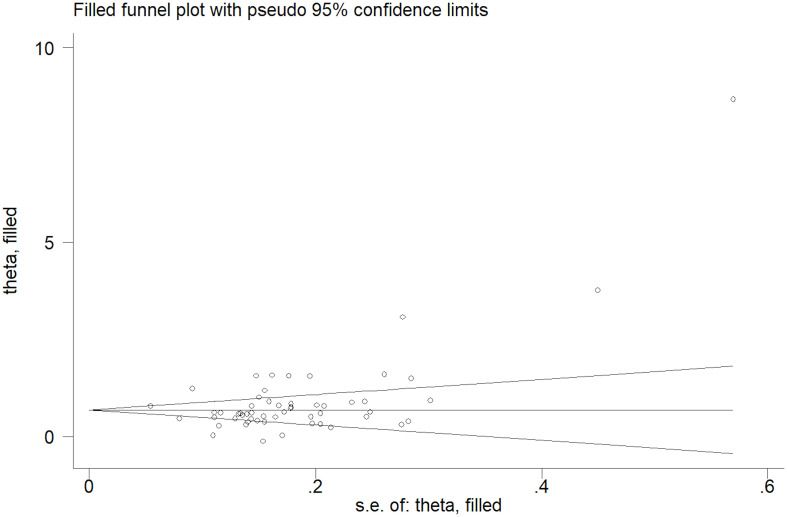
Funnel plot of the association between the SII and rheumatic diseases after “trimming-and-filling”.

Begg’s test suggested funnel-plot asymmetry (p=0.004), whereas Egger’s test did not (p=0.37). The trim-and-fill method did not impute any missing studies. Visual inspection nevertheless indicated that a small number of high-effect estimates contributed to funnel-plot asymmetry (**Figure 4**). In a *post-hoc* sensitivity analysis excluding three such estimates (77, 86, 105), there was no evidence of funnel-plot asymmetry and the pooled effect remained statistically significant (SMD = 0.68, 95% CI 0.58 to 0.79, p<0.001).

### Meta-regression and subgroup analyses

In univariate meta-regression analysis, no significant associations were observed between the effect size and age (t=-0.54, p=0.59), male-to-female ratio (t=-0.36, p=0.72), year of publication (t=0.32, p=0.75), sample size (t=-0.59, p=0.56), mean disease duration (t=-1.25, p=0.23), CRP (t=1.76, p=0.09), ESR (t=1.43, p=0.16), active-to-remission patient ratio (t=-0.37, p=0.71), or DMARD use (t=0.54, p=0.60).

In the subgroup analysis (**Table 2**), the pooled SMD was statistically significant across studies conducted in Asia, Africa, and Europe, with substantially lower heterogeneity in the latter subgroup. Moreover, the pooled SMD was statistically significant across studies of patients with RA, SLE, AS, gout, PsA, AAV, OA, SS, BD, SSc, and FMF, but not in studies of patients with FM or CTD. Virtually no heterogeneity was also observed across studies of patients with SLE, BD, and SSc.

**Table 2:** Subgroup analysis of pooled standardized mean differences (SMD) according to disease type and geographical area.

Disease	Study groups	Effect size			Heterogeneity	
		SMD	95% CI	P-value	I^2^ (%)	P-value
RA	14	0.94	0.68-1.20	<0.001	88.5	<0.001
SLE	9	0.52	0.42-0.63	<0.001	0.0	0.71
AS	7	0.71	0.41-1.01	<0.001	85.3	<0.001
Gout	4	0.56	0.33-0.78	<0.001	80.3	0.002
PsA	3	0.90	0.22-1.58	0.010	92.7	<0.001
AAV	3	3.87	0.67-7.08	0.018	98.6	<0.001
OA	3	0.37	0.01-0.72	0.043	83.5	0.002
SS	3	1.82	0.42-3.23	0.011	96.5	<0.001
FM	3	0.47	-0.34-1.29	0.26	97.3	<0.001
BD	2	0.50	0.23-0.76	<0.001	0.0	0.68
SSc	2	0.85	0.52-1.17	<0.001	0.0	0.74
CTD	1	0.30	-0.24-0.84	0.24	--	--
FMF	1	0.30	0.03-0.57	0.032	--	--
Geographical area	Study groups	Effect size			Heterogeneity	
		SMD	95% CI	P-value	I^2^ (%)	P-value
Asia	46	0.86	0.70-1.01	<0.001	92.7	<0.001
Africa	6	0.79	0.41-1.17	<0.001	84.3	<0.001
Europe	3	0.59	0.25-0.92	0.001	28.5	0.25

AAV, ANCA-associated vasculitis; AS, ankylosing spondylitis; BD, Behcet’s disease; CI, confidence interval; CTD, connective tissue disease; F, female; FM, fibromyalgia; FMF, familial Mediterranean fever; M, male; NR, not reported; OA, osteoarthritis; PsA, psoriatic arthritis; RA, rheumatoid arthritis; SII, systemic immune-inflammation index; SLE, systemic lupus erythematosus; SMD, standardized mean difference; SS, Sjögren’s syndrome; SSc, systemic sclerosis.

### Certainty of evidence

Starting from low certainty because all included studies were observational and cross-sectional, the certainty of evidence was rated as very low (level 1). We made no downgrade for risk of bias or indirectness, downgraded one level for the high and only partly explained heterogeneity, made no downgrade for imprecision, upgraded one level for the large pooled effect size (SMD = 0.83) (46), and downgraded one level for suspected publication bias or small-study effects.

## Discussion

Overall, this meta-analysis demonstrates that SII values are, on average, higher in adults with RDs than in healthy controls, with a large, pooled effect size. The leave-one-out analysis indicates that this result was not dependent on any single study. Nevertheless, the finding should be interpreted as evidence of a group-level difference rather than proof that the SII is a stand-alone diagnostic test. The results therefore support the SII as a candidate adjunctive biomarker that may complement, rather than replace, clinical assessment, autoantibody testing, imaging, CRP, ESR, and validated disease-activity instruments.

The absence of statistically significant associations between effect size and age, sex ratio, publication year, sample size, disease duration, CRP, ESR, active-to-remission ratio, or DMARD use suggests that no single study-level characteristic accounted for the observed association. It is possible that the SII captures inflammatory dimensions that are only partially overlapping with CRP and ESR, which are themselves influenced by age, sex, obesity, infection, liver function, anemia, immunosuppressive therapy, and disease phenotype (22, 26, 112).

The observed direction of effect is biologically plausible. RDs frequently involve the activation of multiple pathways involving innate immune cells, adaptive immune pathways, endothelium, and tissue-resident inflammatory networks. Increased neutrophil counts may accompany granulopoiesis, tissue trafficking, and NET-related autoantigen exposure. Higher platelet counts or platelet activation may reflect cytokine-driven thrombopoiesis and thrombo-inflammatory vascular signaling. Lower lymphocyte counts may occur with immune activation, redistribution, apoptosis, lymphocyte-targeted disease mechanisms, or drug exposure (40–45). The SII captures these changes in a single metric, which may explain why it can identify systemic immune-inflammatory burden even when single inflammatory biomarkers vary across phenotypes.

The subgroup analysis suggests a broad, but not uniform, increase in the SII across the RD spectrum. Significant pooled effects were observed across RA, SLE, AS, gout, PsA, AAV, OA, SS, BD, SSc, and FMF, consistent with systemic inflammation in both autoimmune and autoinflammatory phenotypes. The comparatively large and heterogeneous estimates for AAV and SS should be interpreted with caution because they were based on three study groups and had wide confidence intervals. The non-significant results in FM and CTD also require caution. FM differs mechanistically from inflammatory RDs, whereas the CTD estimate was based on a single heterogeneous group. Although the subgroup analyses support further disease-specific evaluation rather than a single universal SII threshold for all RDs, the observed findings should be considered exploratory given the small number of studies in some subgroups.

The SII was initially studied in patients with liver cancer (113), with subsequent investigations reporting significant associations with clinical outcomes in different types of cancer (36, 114–116), as well as in other disease states (37–39). Studies conducted in patients with atherosclerosis have also reported the potential prognostic superiority of the SII over conventional risk factors (117). Furthermore, in patients with COVID-19, the SII, but not other hematological indices such as the aggregate index of systemic inflammation, the neutrophil-to-lymphocyte ratio, the monocyte-to-lymphocyte ratio, the platelet-to-lymphocyte ratio, and the systemic inflammation response index, was independently associated with adverse outcomes (118). Despite these promising findings, appropriately designed prospective studies are warranted to investigate the diagnostic and prognostic capacity of the SII, alone or in combination with other inflammatory biomarkers and/or clinical parameters, in patients with different types of RDs.

These results extend earlier work on hematological inflammatory indices in immune-mediated disease. Previous reviews have reported higher neutrophil-to-lymphocyte and platelet-to-lymphocyte ratios in RA, SLE, ankylosing spondylitis, psoriasis, and related inflammatory conditions, with inconsistent correlations with disease activity (28–34, 119). The SII differs from these two-component ratios by combining myeloid activation and thrombocytosis in the numerator with lymphocyte counts in the denominator. This may increase sensitivity to a composite inflammatory phenotype, but it also reduces specificity because infections, malignancy, cardiovascular disease, smoking, obesity, pregnancy, recent surgery, corticosteroids, and other stress states can alter one or more of its components.

It is worth noting that the present study is not merely a chronological update of our previous meta-analysis on immunological disorders (47), but rather a substantial expansion and redirection of our research scope. While our earlier work investigated a highly heterogeneous group of immune-mediated conditions based on a limited number of datasets, the current synthesis focuses specifically on RDs. By focusing the analysis on this distinct disease group and substantially expanding the body of literature, we have significantly broadened the scope of interest. This approach enables a comprehensive, dedicated mapping of the SII across a broad and representative spectrum of specific rheumatic phenotypes, many of which were previously unmapped, thereby narrowing confidence intervals and providing significantly more robust, precise, and clinically relevant estimates tailored to the rheumatological field.

The clinical implications of this work are potentially significant as a complete blood count is inexpensive, routinely performed, and available in most healthcare settings, making the SII attractive for screening, triage, and repeated monitoring, particularly where access to specialized immunology assays, musculoskeletal ultrasound, or rheumatology services is limited. However, in addition to the previously discussed lack of diagnostic and prognostic performance data, the SII should not be viewed as a stand-alone diagnostic test for RDs. Its potential role is more likely to be as an adjunctive measure that complements CRP, ESR, autoantibodies, imaging, and validated activity indices. Establishing such use requires studies reporting clinically meaningful thresholds, likelihood ratios, sensitivity, specificity, and predictive values in representative clinical populations.

This study has several strengths. It used a registered protocol, searched three major databases, included a broad range of RDs across the autoinflammatory-autoimmune continuum, applied standardized risk-of-bias and certainty-of-evidence methods, and examined the stability of the pooled estimate through sensitivity analysis. Important limitations include substantial heterogeneity, possibly reflecting differences in RD phenotype, disease activity, disease duration, treatment exposure, and comorbidity exclusion criteria, as well as a lack of evidence from studies conducted in specific geographic regions, particularly in Europe and North and South America. These issues require further study, given the established evidence of differences in inflammatory responses across RD types and ethnic groups (17, 18, 120–123).

Future studies should also move from case-control comparisons to prospective, multicentre, longitudinal designs. Such studies should stratify by RD diagnosis, disease activity, organ involvement, serological status, treatment class, glucocorticoid exposure, comorbidity burden, and ethnicity, and should evaluate whether within-patient changes in the SII are associated with changes in validated activity scores or treatment response. Prediction models should test the incremental value of the SII above CRP, ESR, autoantibodies, complement levels, imaging, and clinical indices.

Taken together, the available evidence indicates that the SII is significantly higher in RD patients than in healthy controls and may provide a low-cost measure of systemic immune-inflammatory burden. However, the substantial heterogeneity observed across studies warrants caution in interpreting the results and calls for further research to identify its sources. Well-designed prospective studies are also needed to determine whether adding the SII to existing clinical, serological, and imaging-based assessments improves diagnosis, disease activity classification, prognosis, or treatment monitoring across specific RDs and patient populations.

## Data Availability

The original contributions presented in the study are included in the article/**Supplementary Material**, further inquiries can be directed to the corresponding author.
