# Supplementary material for: A systematic review and meta-analysis of the systemic immune-inflammation index (SII) in rheumatic diseases
Source: Front Immunol. 2026 Jul 13;17:1896434. doi: 10.3389/fimmu.2026.1896434 (PMC13402447; doi:10.3389/fimmu.2026.1896434)
Supplement: Supplementary file 2 [file Table2.docx]

**Supplementary Table 2.** Assessment of the risk of bias using the Joanna Briggs Institute critical appraisal checklist.

| **Study** | **Were the inclusion criteria clearly defined?** | **Were the subjects and the setting described in detail?** | **Was the exposure measured in a reliable way?** | **Were standard criteria used to assess the condition?** | **Were confounding factors identified?** | **Were strategies to deal with confounding factors stated?** | **Were the outcomes measured in a reliable way?** | **Was appropriate statistical analysis used?** | **Risk of bias** |
| --- | --- | --- | --- | --- | --- | --- | --- | --- | --- |
| Ruta VM et al. | Yes | Yes | Yes | Yes | No | No | Yes | Yes | Low |
| Wu J et al. | Yes | Yes | Yes | Yes | No | No | Yes | Yes | Low |
| Choe JY et al. | Yes | Yes | Yes | Yes | Yes | Yes | Yes | Yes | Low |
| Dincer ABK et al. | Yes | Yes | Yes | Yes | No | No | Yes | Yes | Low |
| Luo Q et al. | Yes | Yes | Yes | Yes | Yes | Yes | Yes | Yes | Low |
| Taha SI et al. | Yes | Yes | Yes | Yes | No | No | Yes | Yes | Low |
| Amirpour A et al. | Yes | Yes | Yes | Yes | Yes | Yes | Yes | Yes | Low |
| Chikovani T et al. | Yes | Yes | Yes | Yes | No | No | Yes | Yes | Low |
| Choe JY et al. | Yes | Yes | Yes | Yes | No | No | Yes | Yes | Low |
| Dede BT et al. | Yes | Yes | Yes | Yes | No | No | Yes | Yes | Low |
| Jiang Y et al. | Yes | Yes | Yes | Yes | Yes | Yes | Yes | Yes | Low |
| Karadeniz H et al. | Yes | Yes | Yes | Yes | No | No | Yes | Yes | Low |
| Ozdemir A et al. | Yes | Yes | Yes | Yes | No | No | Yes | Yes | Low |
| Sariyildiz A et al. | Yes | Yes | Yes | Yes | No | No | Yes | Yes | Low |
| Sugimoto E et al. | Yes | Yes | Yes | Yes | No | No | Yes | Yes | Low |
| Tarabeih N et al. | Yes | Yes | Yes | Yes | Yes | Yes | Yes | Yes | Low |
| Akdogan MR et al. | Yes | Yes | Yes | Yes | No | No | Yes | Yes | Low |
| Başaran PO et al. | Yes | Yes | Yes | Yes | Yes | Yes | Yes | Yes | Low |
| Dervisevic A et al. | Yes | Yes | Yes | Yes | No | No | Yes | Yes | Low |
| Elnemr RA et al. | Yes | Yes | Yes | Yes | No | No | Yes | Yes | Low |
| Ergun MC et al. | Yes | Yes | Yes | Yes | No | No | Yes | Yes | Low |
| Kılıc O et al. | Yes | Yes | Yes | Yes | Yes | Yes | Yes | Yes | Low |
| Misirci S et al. | Yes | Yes | Yes | Yes | No | No | Yes | Yes | Low |
| Okutan I et al. | Yes | Yes | Yes | Yes | No | No | Yes | Yes | Low |
| Rabrenovic V a et al. | Yes | Yes | Yes | Yes | No | No | Yes | Yes | Low |
| Sariyildiz A et al. | Yes | Yes | Yes | Yes | No | No | Yes | Yes | Low |
| Uzeli US et al. | Yes | Yes | Yes | Yes | Yes | Yes | Yes | Yes | Low |
| Zhang Y et al. | No | Yes | Yes | No | No | No | Yes | Yes | Moderate |
| Aci R et al. | Yes | Yes | Yes | Yes | No | No | Yes | Yes | Low |
| Baran E et al. | Yes | Yes | Yes | Yes | No | No | Yes | Yes | Low |
| Ozdogan Bircan A et al. | Yes | Yes | Yes | Yes | No | No | Yes | Yes | Low |
| Dogan M et al. | Yes | Yes | Yes | Yes | No | No | Yes | Yes | Low |
| Ecesoy V et al. | No | Yes | Yes | Yes | No | No | Yes | Yes | Moderate |
| Gunaydin EB et al. | Yes | Yes | Yes | Yes | No | No | Yes | Yes | Low |
| Helbawi FM et al. | Yes | Yes | Yes | Yes | No | No | Yes | Yes | Low |
| Huang C et al. | Yes | Yes | Yes | Yes | Yes | Yes | Yes | Yes | Low |
| Kelesoglu Dincer AB et al. | Yes | Yes | Yes | Yes | No | No | Yes | Yes | Low |
| Klisic A et al. | Yes | Yes | Yes | Yes | No | No | Yes | Yes | Low |
| Koca N et al. | Yes | Yes | Yes | Yes | No | No | Yes | Yes | Low |
| Kosehasanogullari M et al. | Yes | Yes | Yes | Yes | Yes | Yes | Yes | Yes | Low |
| Misirci S et al. | Yes | Yes | Yes | Yes | No | No | Yes | Yes | Low |
| Rapapa KA et al. | Yes | Yes | Yes | Yes | Yes | Yes | Yes | Yes | Low |
| Uysal A et al. | Yes | Yes | Yes | Yes | No | No | Yes | Yes | Low |
| Wu H et al. | Yes | Yes | Yes | Yes | No | No | Yes | Yes | Low |
| Yang CH et al. | Yes | Yes | Yes | Yes | Yes | Yes | Yes | Yes | Low |
| Yigit E et al. | Yes | Yes | Yes | Yes | Yes | Yes | Yes | Yes | Low |
| Yu R et al. | Yes | Yes | Yes | Yes | No | No | Yes | Yes | Low |
| Zhao H et al. | Yes | Yes | Yes | Yes | No | No | Yes | Yes | Low |
| Abdul-Sahib NS et al. | Yes | Yes | Yes | Yes | No | No | Yes | Yes | Low |
| Ashour DM et al. | Yes | Yes | Yes | Yes | No | No | Yes | Yes | Low |
| Li M et al. | Yes | Yes | Yes | Yes | Yes | Yes | Yes | Yes | Low |
| Tuzun Z et al. | Yes | Yes | Yes | Yes | No | No | Yes | Yes | Low |
| Zhang J et al. | Yes | Yes | Yes | Yes | No | No | Yes | Yes | Low |
